# Comparison of the Efficacy of Continuous Femoral Nerve Block With Epidural Analgesia for Postoperative Pain Relief After Unilateral Total Knee Replacement

**DOI:** 10.7759/cureus.24524

**Published:** 2022-04-27

**Authors:** Muhammad Nafees-ul Hasan, Salman A Saleem, Shafi-ur Rehman Rao, Muhammad Hasan Wasim, Naveed A Durrani, Sidra A Naqvi

**Affiliations:** 1 Pain Management, Shifa International Hospital Islamabad, Islamabad, PAK; 2 Anaesthesiology, Shifa International Hospital Islamabad, Islamabad, PAK

**Keywords:** total knee replacement (tkr), pain management, numeric pain rating scale, femoral nerve block, epidural injections

## Abstract

Introduction

With recent developments in postoperative pain management after total knee replacement (TKR), the continuous femoral nerve block is becoming a common practice. The purpose of this study was to compare a femoral nerve block with time-tested epidural analgesia in a tertiary care setup in a developing country.

Methodology

A randomized control trial took place at Shifa International Hospital (SIH), Islamabad, Pakistan. Sixty patients, aged 40 to 90 years old, 12 males and 48 females, who were undergoing unilateral TKR for osteoarthritis in American Society of Anesthesiologists (ASA) physical status classes I and II, weighing between 50 and 99 kg, and fully able to understand and respond to the numeric rating scale (NRS) were included in the study. While patients belonging to ASA physical status class ≥3, with chronic opiate therapy, having allergies to local anesthetics or equipment material, or with neuromuscular disease, were excluded from the study. Ethical approval was obtained, and patients were divided into two groups, with group A given epidural and group B given a femoral nerve block for pain management postop. Data were collected. The pain was recorded using the NRS at six, 12, and 24 hours postop.

Results

The results for six hours and 12 hours were found to be significant. Patients in group A had a lower NRS rating postop as compared to group B and required a lesser amount of additional boluses for pain management.

Conclusion

The femoral nerve block is inferior to epidural analgesia for pain management after unilateral TKR in the first 24 hours, with a greater need for extra boluses to relieve pain.

## Introduction

Total knee replacement (TKR) is a commonly performed procedure, with up to one million TKRs performed per year in the US. It is expected to increase to 3.5 million per year in 2030 [1]. This procedure is associated with significant postoperative pain. Postoperative pain relief is a major determinant of a patient’s satisfaction with treatment in addition to the physiological ill effects. Postoperative pain relief is also important because inadequately treated acute pain may lead to chronic pain [2].

Analgesic options after TKR include pre-emptive analgesia, local infiltration, systemic analgesics (opioids, non-opioids, and patient-controlled analgesia), epidural analgesia, nerve blocks, and multimodal approaches [3]. Among all, epidural analgesia has turned out to be the most widely used analgesia technique in routine clinical practice involving knee replacement surgeries. This technique reduces neuroendocrine stress responses, central sensitization of the nervous system, and muscle spasms that occur in response to painful stimuli [4]. The side effects of epidural analgesia include some motor blockade, dysfunction of bowel and bladder activity, and hypotension [5]. The femoral nerve is one of the nerves that supply the knee joint [6]. An alternative to epidural analgesia is peripheral nerve blockade of the femoral nerve using catheters for continuous infiltration, also called continuous perineural blockade (CPNB). Apparently, this avoids motor block of the contralateral leg, hemodynamic instability of sympathectomy, and bowel and bladder dysfunction.

In 2017, Vishwanatha and Kalappa compared continuous femoral nerve blockade (CFNB) with epidural analgesia for postoperative pain relief in knee surgeries [7]. Moreover, Lu, Huang, and Yan compared femoral nerve block, epidural block, and intravenous patient-controlled analgesia (PCA) in pain control after TKR [8]. All these studies concluded that CFNB provides postoperative analgesia equivalent to that obtained with epidural analgesia but with fewer side effects.

At our facility, only epidural analgesia has been used for postoperative pain relief for bilateral as well as unilateral TKR. Inspired by the above-mentioned studies, we carried out a randomized controlled trial to compare the efficacy of continuous femoral nerve block with continuous epidural analgesia for postoperative pain relief in the first 24 hours.

## Materials and methods

This study was a randomized control trial (RCT). The trial was started after the approval from the Ethical Board of Shifa International Hospital and was conducted at Shifa International Hospital. It was conducted over a period of six months. Patients, aged 40 to 90 years, scheduled for unilateral TKR for osteoarthritis, belonging to the American Society of Anesthesiologists (ASA) physical status classes I and II, weighing between 50 and 99 kg, and fully able to understand and respond to the NRS were included in the study [9]. Patients belonging to ASA physical status class ≥3, on chronic opiate, having allergies to local anesthetics or equipment material, or with neuromuscular disease were excluded from the study. Patients who fulfilled the inclusion criteria for the study were first presented in the pre-anesthesia clinic and counseled by a consultant anesthetist regarding procedures of both techniques and the use of NRS (i.e on a scale from 0 to 10, 0 = no pain and 10 = worst possible pain).

Patients were divided into two groups using consecutive random sampling. The patients in group A, the epidural group, received combined spinal-epidural anesthesia in the L3-L4 interspace in the lateral decubitus position with the operative site on the dependent side. For surgery, spinal anesthesia with 2 ml of 0.75% bupivacaine and 20 ug of fentanyl was used. The patients in group B, the femoral nerve block group, received intrathecal anesthesia with the same drugs in the lateral decubitus position with the operative site on the dependent side. After turning supine, an ultrasound-guided femoral nerve block catheter was passed under aseptic conditions on the operative side. Surgery was completed under intrathecal anesthesia. In the post-anesthesia care unit (PACU), infusion of bupivacaine 0.187 % was started in both groups at 8-10 ml/hour through epidural and femoral catheters. The patient received an injection of paracetamol 1000 mg intravenous every six hours, an injection of ketorolac 30 mg every eight hours, and a capsule of pregabalin 25 mg every 12 hours regularly during the postoperative period. Injection tramadol 50 mg was given for rescue to the patients only if the pain score exceeded 4 on NRS every six hours with 25 mg of injection Gravinate. The pain was assessed at six, 12, and 24 hours by a pain fellow on duty using NRS and documented on the proforma along with the total number of rescue tramadol.

The results were averaged (mean ± standard deviation) for continuous data as mentioned in the results section. The Mann-Whitney U-test was applied to find out the significant difference between the two independent groups. In the entire above test, P-value < 0.05 was accepted as indicating statistical significance. Data analysis was carried out using Statistical Product and Service Solutions (SPSS version 26; IBM Corp. Armonk, NY).

## Results

A sample size of 62 patients was recruited in the current study. This was done to avoid any untoward dropout because of the coronavirus disease 2019 (COVID-19) pandemic. Two patients dropped out. One patient was diagnosed as COVID positive and the second patient had financial issues. So 60 patients were treated, with a minimum age of 42 and maximum age of 81. The mean age of the total participants was 62.82 ± 8.25. Patients were equally divided into two groups (group A and group B) by consecutive random sampling. Each group contained 30 patients. Forty-eight females and 12 males were recruited for this study.

Intergroup analysis was carried out using the Mann-Whitney U-test (non-parametric) (Table 1). The results showed a significant difference for NRS scoring at six hours and 12 hours. Patients who were in group A and were given Epidural postop for pain management showed better results and lower values for NRS and needed fewer extra boluses for their pain management while, on the other hand, group B patients who were given a femoral nerve block for pain management showed higher NRS scores and needed more extra boluses for relieving their pain (Table 2).

**Table 1:** Mann-Whitney U-test scoring for NRS six, 12, and 24 hours postop NRS = numerical rating scale

Variables	Mean Rank		P-value
	Group A (Epidural)	Group B (Femoral nerve block)	
NRS 6 hours postop	18.18	42.82	.000
NRS 12 hours postop	16.70	44.30	.000
NRS 24 hours postop	28.63	32.37	.356

**Table 2:** Number of boluses needed postop by patients to manage pain

		Bolus					Total
		.00	1.00	2.00	3.00	4.00	
Type of analgesia given	Epidural	2	15	5	6	2	30
	Femoral nerve block	5	0	0	8	17	30
Total		7	15	5	14	19	60

## Discussion

This study shows that a continuous femoral nerve block is inferior to epidural analgesia at six and 12 hours after unilateral TKR in terms of analgesia. The benefits of epidural analgesia and surgical outcomes have been well-documented in the literature. Nowadays, specific pathways of care, including enhanced recovery after surgery (ERAS), have been introduced. It has been proposed that these pathways, along with multimodal analgesia, have reduced the length of hospital stay (LOS) for patients undergoing TKR [10]. Peripheral nerve blocks like femoral and sciatic nerve blocks have been the components of multimodal analgesia. Most commonly, a femoral nerve block has been propagated for postoperative analgesia after TKR [7-8]. It has been postulated that peripheral nerve blocks (PNBs) provide intense, site-specific analgesia and are associated with a lower incidence of side effects when compared with many other modalities of postoperative analgesia [11]. Continuous catheter techniques provide extended analgesia and improved functional recovery. These result in shorter hospital stays [12]. Regarding TKR, only unilateral surgery can be an indication of the nerve block technique. Hence, we compared epidural analgesia with femoral nerve block in unilateral TKR while keeping other elements of multimodal analgesia constant.

However, this study failed to prove that a femoral nerve block is equivalent in analgesic efficacy to epidural analgesia (p-value<0.05 at 6 and 12 hours). This is fairly understandable when we look at the nerve supply of the knee joint. Innervation of the knee is complex, with contributions from both the lumbar and sacral plexuses. It follows Hilton's law, i.e. nerves supplying the muscles around a joint supply the joint as well [13]. This means that the femoral, obturator, tibial, and peroneal nerves all have contributions to the nerve supply of the knee joint. If we target only one nerve, i.e. the femoral nerve, we can't expect perfect analgesia because contributions from other nerves remain unaddressed. This is especially true in the early postoperative period, i.e. the first 24 hours when pain is maximum. This was also evident in earlier studies that showed higher pain scores in the first 24 hours in femoral nerve block groups [7-9]. Later in the course, pain scores became comparable in both groups. Likewise, in our study, pain scores at six and 12 hours postoperatively were significantly high in the femoral nerve block group. At 24 hours, both modalities became equal in analgesic efficacy. Also, the use of opioid rescue analgesia was more in the femoral nerve block group as compared to the epidural group, with more episodes of nausea and vomiting requiring additional antiemetics. As evident from Table 2, 17 patients in the femoral nerve block group received four boluses of tramadol in the first 24 hours while only two patients received four boluses of tramadol in the epidural group. Five patients in the femoral nerve block group had to be put on PCA morphine because pain scores remained persistently at or above 4 despite rescue analgesics and routine medicines.

However, as the level of significance was set at 80%, the sample size was small. This limits the results of the study. Second, five patients in the femoral nerve block group were excluded from the study, further reducing the sample size. Third, operator expertise with an ultrasound-guided femoral nerve block can be a limitation of the study.

## Conclusions

This study shows that a femoral nerve block is inferior in efficacy to epidural analgesia for postoperative pain relief after unilateral TKR. The femoral nerve block leads to higher pain scores and greater opioid requirements postoperatively. Further research is needed to investigate whether combining it with sciatic nerve block or patient-controlled analgesia (PCA) morphine can improve pain scores or not. Until then, epidural analgesia should be continued as a routine postoperative pain management tool after unilateral TKR.
